# Always one step ahead: How pathogenic bacteria use the type III secretion system to manipulate the intestinal mucosal immune system

**DOI:** 10.1186/1476-9255-8-11

**Published:** 2011-05-03

**Authors:** Anna Vossenkämper, Thomas T MacDonald, Olivier Marchès

**Affiliations:** 1 Centre for Immunology and Infectious Disease, Blizard Institute of Cell and Molecular Science, Barts and the London School of Medicine and Dentistry, London, UK

**Keywords:** gut-associated lymphoid tissue, type 3 secretion system, EPEC, Shigella

## Abstract

The intestinal immune system and the epithelium are the first line of defense in the gut. Constantly exposed to microorganisms from the environment, the gut has complex defense mechanisms to prevent infections, as well as regulatory pathways to tolerate commensal bacteria and food antigens. Intestinal pathogens have developed strategies to regulate intestinal immunity and inflammation in order to establish or prolong infection. The organisms that employ a type III secretion system use a molecular syringe to deliver effector proteins into the cytoplasm of host cells. These effectors target the host cell cytoskeleton, cell organelles and signaling pathways. This review addresses the multiple mechanisms by which the type III secretion system targets the intestinal immune response, with a special focus on pathogenic *E. coli*.

## Review

### The gut-associated lymphoid tissue

The intestinal lumen is exposed to the environment and therefore in continuous contact with harmless as well as pathogenic microorganisms. Thus, it is not surprising that the gut is the biggest lymphoid organ in the body and contains about 70% of the body's immune cells [1-3]. The gut-associated lymphoid tissue (GALT) uses a range of mechanisms to protect the host from pathogens, while it at the same time tolerates commensal microorganisms. Furthermore, the GALT needs to prevent the invasion of harmful agents without affecting the absorption of nutrients from the lumen.

**Figure 1:**
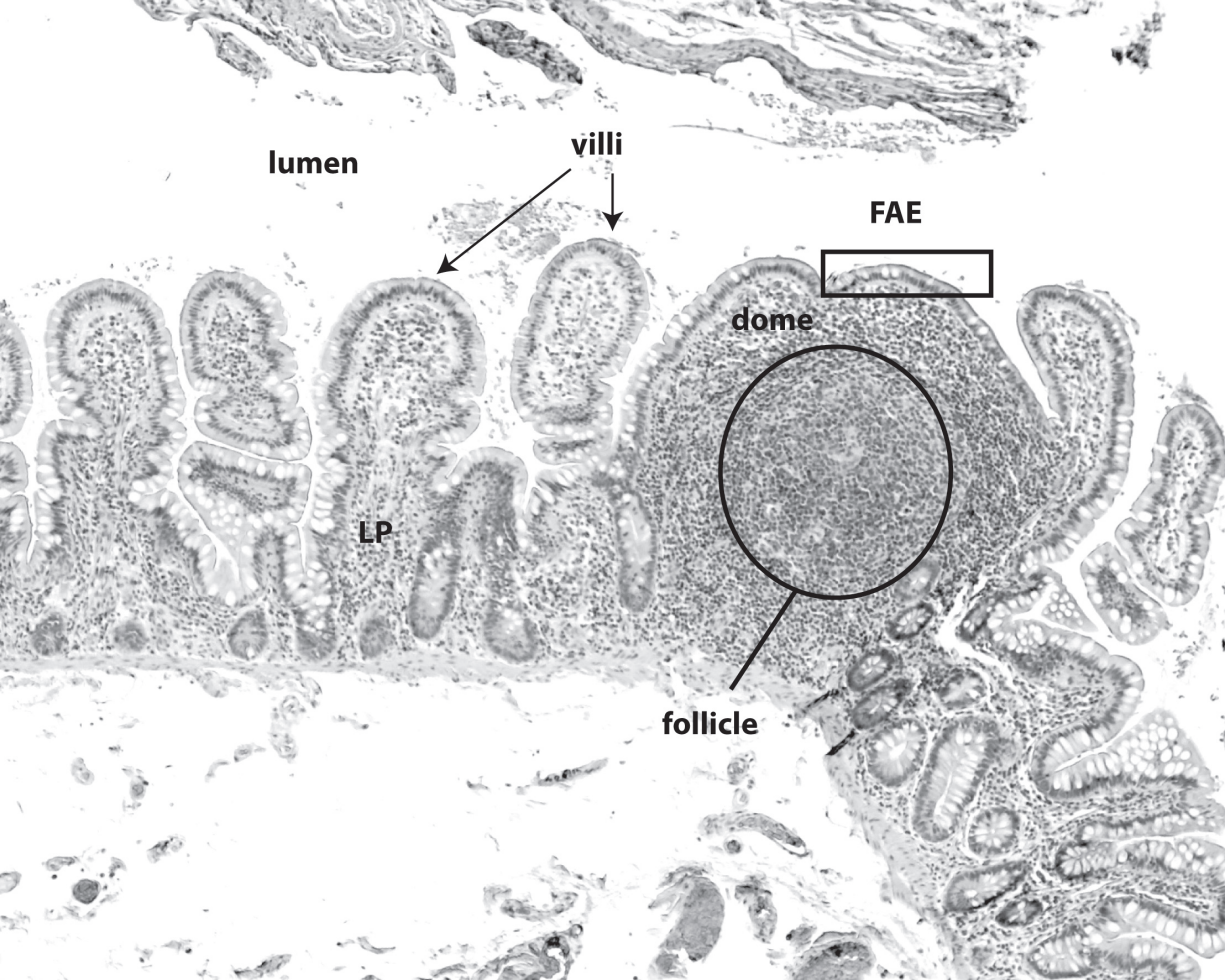
**Follicles and PP are the inductive site for the mucosal immune response**. Micrograph of a human ileal lymphoid follicle stained with hematoxylin & eosin. The follicle is covered by M-cells which form the follicle-associated epithelium (FAE). Underneath the dome area which holds dendritic cells, is a B cell follicle, surrounded by a T cell-rich zone. Adjacent to the follicle are microvilli. LP = lamina propria.

GALT is comprised of the appendix, single lymphoid follicles (Figure 1) in the small and large intestine, and the Peyer's patches (PP). The latter are clusters of follicles and have a distinct architecture with germinal centers containing B cells and follicular dendritic cells (DCs) which are surrounded by areas with T cells and macrophages [2]. PP are covered by specialized micro-folded epithelial cells, the M-cells, which make up the follicle-associated epithelium (FAE). This epithelium forms the interface between the luminal microorganisms and the immune cells of the GALT [4]. PP have no afferent lymph vessels and antigens are received directly from the intestinal lumen. After the uptake of luminal material by endocytosis and phagocytosis, the M-cells deliver antigens and microbes to antigen-presenting cells in the subepithelial dome of the PP, which subsequently present them to PP T cells [2]. PP DCs also directly sample bacteria from the intestinal lumen by sending protrusions through the epithelial layer without disrupting epithelial integrity [5]. Therefore, follicles and PP are an inductive site where microorganisms are sensed and the appropriate immune response is initiated [4]. In contrast, the lamina propria is an effector site; after activation in PP, DCs migrate to the mesenteric lymph nodes where they present antigens to B and T cells and the immune response is amplified. *Via *expression of homing molecules, mainly the integrin alpha4beta7 and CCR9, the lymphocytes are then able to re-enter the mucosal site where they contribute to immune defense along the entire length of the intestine [6].

The gut has a wide range of strategies to fight infections. Amongst the non-specific mechanism are the mucus layer which traps microorganisms, the secretion of anti-microbial agents such as defensins, trefoil factors and proteases, intestinal peristalsis, and the natural microbiota which compete with pathogens for epithelial binding and nutrients [7-10]. Microorganisms are also largely prevented from epithelial attachment by secretory IgA (sIgA) which binds them in the lumen and mucus [11]. Germinal center B cells receive multiple activation and survival signals from follicular DCs in PP upon encounter with bacterial products leading to the generation of IgA secreting plasmablasts [12]. After activation and T cell-dependent class switching to IgA in the PP, B cells eventually migrate to the lamina propria where they reside as IgA secreting plasma cells [13,14]. Cellular defense mechanisms within the lamina propria are crucial in reducing and dealing with invasion of pathogens. The main cell population comprises CD4+ T lymphocytes which, depending on the cytokine milieu, respond by producing factors associated with a Th1 immune response which is crucial for the response to intracellular pathogens and stimulates phagocytosis by macrophages. A Th2 response is typically established following infection with parasites and involves production of IL-4, IL-5, IL-10, and IL-13 resulting in activation and recruitment of B cells, mast cells and eosinophils [15].

The lamina propria also contains natural killer cells which are thought to mediate intestinal homeostasis by producing IL-22 and exhibit their cytotoxic functions upon activation by T cells [16,17]. IL-22 has been shown to be an important factor in the host defense against enteral bacteria, like e.g. *Citrobacter rodentium*, a murine pathogen which is used to study infections with enteropathogenic *E. coli *(EPEC) and enterohaemorrhagic *E. coli *(EHEC) in humans [18]. Additional studies highlighted the importance of IFNg-producing CD4+ T cells in the defense against these bacteria [19].

Another layer of defense is located in the epithelium where a large population of mainly CD8αβ intraepithelial T lymphocytes resides in the basolateral area, between the epithelial cells [20]. Some studies demonstrated cytolytic activity of these T cells which suggest they might be involved in cancer surveillance and killing of infected cells [21].

### Recognition of bacteria in the intestine

The intestinal immune system faces the constant challenge of discriminating between the commensal microbiota and pathogens. The response to the latter is usually rapid and results in the activation of innate and adaptive immune mechanisms that lead to inflammation and eradication of the pathogen, sometimes with considerable damage to the intestinal mucosa. Non-pathogenic bacteria that form the microbiota are also recognized by the GALT [22]; however, the immune response to commensals appears to be strictly controlled, and does not lead to overt inflammation. How the GALT discriminates between these two categories of microorganisms, commensals and pathogens, is complex and not fully understood. However, DCs in GALT are of paramount importance for responding to bacterial stimuli and the initiation of a tolerogenic state by promoting the expression of anti-inflammatory molecules like IL-10 and TGFbeta [23].

In the last two decades, with the discovery of toll-like receptors (TLR) and Nod-like receptors (NLR) which recognize pathogen-associated molecular patterns (PAMPs), the knowledge about the recognition of microbial structures by immune and epithelial cells has dramatically increased [24]. These receptors specifically bind ligands widely shared amongst pathogens. Well-characterised examples of such ligands are bacterial cell wall components such as peptidoglycans and lipoproteins (both binding to TLR2) or nucleic acid ligands such as bacterial CpG DNA which binds to TLR9 [25,26]. Immune recognition *via *pattern-recognition receptors is crucial for host defense and immune homeostasis, and dysfunction of these receptors has been shown to be associated with gut inflammatory conditions such as Crohn's disease [27]. Binding of microbial ligands to TLRs (besides TLR3) results in the activation of a pro-inflammatory MyD88-dependent pathway that leads to activation of the transcription factor NF-kappaB. Another signaling pathway that is critically involved in inflammation is the mitogen-activated protein (MAP) kinase-cascade. Although not activated by microbial ligands, this pathway is initiated by extracellular stimuli like pro-inflammatory cytokines or mitogens [28]. Both the NF-kappaB and MAPK pathway are activated in intestinal infections by pathogens which use type III secretion systems (T3SS) [29]. These pathways are also amongst the known targets for T3SS effectors. An overview on how these pathways are affected during intestinal infection with pathogens that employ the T3SS is discussed here, with special emphasis on EPEC and EHEC. These two pathogens, also known as attaching and effacing pathogens (A/E), are amongst the leading causes for diarrheal diseases. EPEC is a big health concern, especially for infants, in developing countries. An EPEC infection can be asymptomatic, but the classical feature of the infection is profuse watery diarrhea in combination with vomiting. EHEC is responsible for food-borne outbreaks of diarrheal diseases, with contaminated beef being the most common vehicle for infection. Certain EHEC strains (e.g. O157:H7) produce Shiga-like toxins which can cause potentially life threatening complications like the hemolytic-uremic syndrome (HUS). This disease is characterized by acute kidney failure, thrombocytopenia and hemolytic anemia and affects mostly children. The mortality of HUS is approximately 5-10% and it is therefore a medical emergency requiring intensive clinical care.

### Sustaining colonization by preventing bacterial detachment and death of infected cells

Some of the most successful gram-negative pathogens use the type 3 secretion system (T3SS), a molecular syringe, to inject an arsenal of virulence effector proteins directly into the cytoplasm of the host cells. The effectors can then target and hijack various host cell functions for the benefit of the pathogen [30]. The increasing understanding of the variety of T3SS effectors and their functions has given rise to the idea that for every defense strategy used by the host, there might be antagonistic effector proteins. Recent data gained from research on the function of *Shigella *effectors, illustrate this hypothesis [31]. One of the protective mechanisms of the gut mucosa is the constant renewal and shedding of epithelial cells at the top of the villi in the small bowel and from the colon surface. If subjected to bacterial colonization, the enterocytes can undergo programmed cell death and detach from the extracellular matrix into the lumen, preventing the pathogen crossing the epithelium [32,33]. *In vitro *and *in vivo *data identified two *Shigella *effectors, IpaB and OspE, which counteract the intestinal epithelial turnover and exfoliation [34,35]. IpaB causes a cell cycle arrest of infected cells by interacting with Mad2L2, an inhibitor of the anaphase promoting complex (APC) which regulates the cell cycle [34]. In a rabbit ileal loop model, intestinal crypts infected with *Shigella *that express an IpaB mutant protein which is unable to interact with Mad2L2, have a higher number of progenitor cells and are less colonized than with the wild type strain. These findings suggest that IpaB, by blocking intestinal cell proliferation and renewal, prolongs *Shigella *colonization [34]. *Shigella *also injects the effector OspE into enterocytes which stabilizes the adhesion of intestinal cells to the extra-cellular matrix by targeting and modulating the function of integrin-linked kinase (ILK), a modulator of focal adhesion [35]. The interaction between OspE and ILK enhances the presence of beta1-integrin at the cell surface and prevents the disassembly of focal adhesions. An *in vivo *study performed in a guinea pig colon infection model showed reduced colonization and pathogenicity of OspE mutant bacteria. This study suggests that OspE enhances the infectivity of *Shigella *by preventing the exfoliation of infected intestinal cells [35].

Some EPEC and EHEC strains as well as the mouse pathogen *Citrobacter rodentium *might also use a similar strategy as they possess the effector EspO which has strong homology with *Shigella*'s OspE [35,36]. The inhibition of epithelial cell detachment is an emerging theme in bacterial pathogenesis, and recent *in vivo *work suggests that it is a strategy shared by all bacteria that are able to bind human carcino-embryonic antigen-related cell adhesion molecules (CEACAM), e.g. *Neisseria gonorrhoeae, Neisseria meningitidis, Moraxella catarrhalis*, and *Haemophilus influenzae *[32,37].

Interestingly, some EPEC strains produce the effector Cif which blocks the cell cycle of infected cells [38]. Cif binds Nedd8, a ubiquitin-like protein and inhibits neddylated Culling-RING ligases-induced (CLRs) ubiquitination of a variety of CLR substrates, such as the cell cycle inhibitors p21waf1 and p27kip1 [39,40]. It is thus possible that in EPEC, Cif acts like *Shigella*'s IpaB, and also prolongs colonization of the gut mucosa by preventing epithelial cell renewal.

Other work suggests that inhibition of the epithelial renewal and exfoliation could indeed be an infective strategy of EPEC and EHEC pathogens. Shames and co-workers have demonstrated a role for the effector EspZ in reducing the death and detachment of epithelial cells infected with EPEC *in vitro *[41]. EspZ binds the transmembrane glycoprotein CD98 and enhance its effect on beta1-integrin signaling and cell survival *via *activation of focal adhesion kinase. EspZ also activates the pro-survival AKT pathway, which does not seem to rely on CD98 binding [41].

A potent pro-survival activity has also been identified for NleH1 and NleH2, two effectors produced by EPEC and EHEC. NleHs inhibits apoptosis *via *various stimuli in epithelial cells, dependent on the binding to the anti-apoptotic Bax inhibitor-1 [42]. The mechanism by which NleH prevents cell death is independent of its kinase function and remains to be determined.

**Figure 2:**
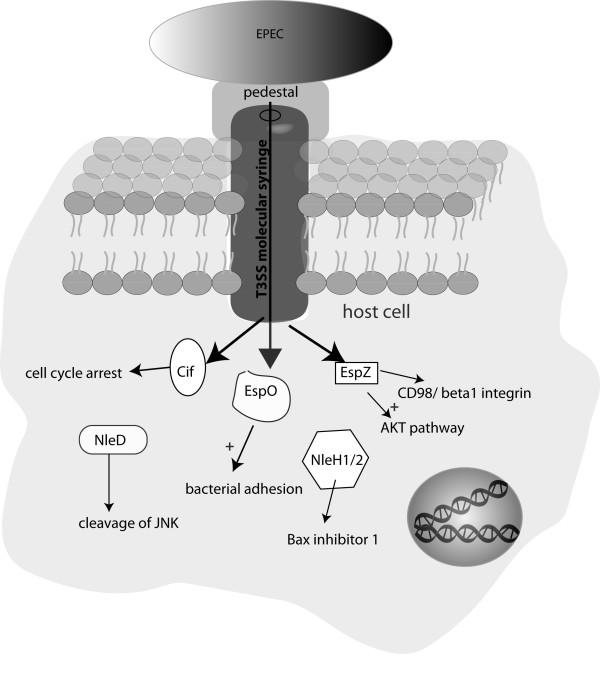
**EPEC uses several effector proteins to promote bacterial adhesion**. After binding to the epithelial cell, EPEC uses the T3SS to inject effectors into the host cell cytoplasm *via *a needle-like structure. Intimate adhesion to the host cell is secured by rearrangement of the actin cytoskeleton and the formation of a pedestal. Amongst the injected effectors are EspO, NleH1, NleH2, EspZ, and CiF which modulate the cell cycle and apoptotic regulation, resulting in reduced epithelial renewal and prolonged bacterial adherence.

Another effector reported to be potentially involved in anti-apoptotic activity is the metalloprotease NleD which prevents JNK-mediated pro-apoptotic signaling by cleaving and inactivating JNK [43]. Apart from the effector EspZ, which is severely attenuated for virulence in the mouse model [44], an essential role for other effectors in virulence like NleHs and NleD have not been established in different animal models [45,46]. Although *in vivo *evidence is missing, these studies suggest that EPEC and EHEC use EspO, EspZ, NleH, and NleD to prevent or delay the exfoliation and apoptotic clearance of the targeted cells in the intestinal epithelium and to sustain bacterial colonization (Figure 2 and table 1).

**Table 1:** Effectors of EPEC/EHEC that modulate cell detachment, pro-inflammatory signaling, and phagocytosis

Effector	Cellular targets^a^	Biochemical activity/characteristics^b^	Phenotype	*In vivo *role
**Inhibition of cell detachment and modulation of cell death**				

**NleH1** **NleH2**	Bax inhibitor-1 (BI-1)	Binds to N-terminal amino acid 1-40 of BI-1. N-terminal aa 1-100 of NleHs not required for binding to BI-1	Inhibition of apoptosis induced *via *multiple stimuli	Various roles reported *in vivo*. NleH reduces the level of apoptotic colonic cells in mouse model [42]

**EspZ**	CD98	C-terminal amino acid domain 43-99 required for CD98 binding	Prevent cell detachment. Enhance activation of pro-survival FAK and AKT pathway. Binding to CD98 promotes β1-integrin activation of FAK.	Mutant *espZ *attenuated for colonization and hyperplasia in mice [44]

**NleD**	JNKs, p38	Zinc metalloprotease (motif _142_HExxH_146_)	Cleaves MAP kinases JNK and p38 in the activation loop. Reduce JNK pro-apoptotic activity	Enhance colonization in calves, no role identified in mice and lamb infection models [45,46]

**Cif**	NEDD8	Deamidase of NEDD8 and ubiquitin	Block cell cycle at G2/M and G1/S transitions [39]	Unknown

**EspO/** **OspO**	ILK (?)	*Shigella*'s OspE C-terminal _68_W essential for activity is conserved in EPEC/EHEC EspO/OpsO	Prevent cell detachment?	Unknown

**Inhibition of pro-inflammatory signaling**				

**NleE**	Unknown	C-terminal _208_IDSYMK_214 _motif essential for activity	Inhibits TNFα, IL-1β and PRRs mediated activation of NF-kappaB and expression of pro-inflammatory cytokines in epithelial and immune cells. Acts by inhibition of IκBα phosphorylation blocking p65 nuclear translocation	Slight role in colonization and persistence reported [45,60]

**NleC**	p65, p50, c-Rel, IκBα	Zinc metalloprotease (motif _183_HExxH_187_) N-terminal domain aa 33-64 required for p65 and p50 binding	Cleaves p65 and p50 to inhibit NF-kappaB activation. Cleavage of c-Rel and IκBα also reported.	No role identified in mice and lamb infection model [45,46]

**NleB**	Unknown	Unknown	Inhibit TNFα-mediated NF-kappaB activation	Required for colonization and disease in mouse model [45,60]

**NleH1**	Ribosomal protein S3 (RPS3)	Activity in N-terminal 139 amino acid (N40 and K45 required for RPS3 inhibition)	Prevent RPS3 nuclear translocation and expression of RPS3-NF-kappaB dependent pro-inflammatory genes	NleH1 EHEC mutant hypervirulent in piglet infection model [55]

**NleH1 and** **NleH2**	Unknown	Serine-threonine kinase motif	Prevent IκBα ubiquitination and degradation	Required for colonization and reduction of inflammation in EPEC mouse model [58]

**NleD**	JNK, p38	Zinc metalloprotease (motif _142_HExxH_146_)	Cleaves MAP kinases JNK and p38 in the activation loop. Contributes to overall bacterial mediated inhibition of IL-8 *in vitro*.	Mutant not attenuated in mice, calve and lamb models [45,46]. Role in colonization in STM screen in calves

**Inhibition of phagocytosis**				

**EspF**	Unknown	N-term 101 amino acid for anti-phagocytic activity	Prevents PI3K-dependent phagocytosis of bacteria; Reduces uptake of EPEC bacteria in *in vitro *M cell model	EspF mutant attenuated in mice model. Specific role of anti-phagocytic activity unknown [77,78]

**EspB**	Myosin proteins	Domain from amino acid 159-218 essential for myosin binding	Prevents bacterial phagocytosis *via *inhibition of myosin-actin interaction	*Citrobacter *expressing EspB mutated for myosin binding are attenuated in mouse model [74]

**EspJ**	Unknown	Unknown	Blocks FcγR and CR3-opsonophagocytosis	Role in bacterial clearance reported in mouse model [75]

**EspH**	RhoGEFs	Binds to DH-PH domain of RhoGEFs and inhibits RhoGTPase signalling	Attenuates bacteria phagocytosis and FcγR-mediated phagocytosis	EspH mutant not or slightly attenuated for colonization in mice and rabbit model [44]

^a ^In relation to phenotype described; ^b ^Motif or biochemical activity required for phenotype described

### Modulation of proinflammatory signaling pathways

**Figure 3:**
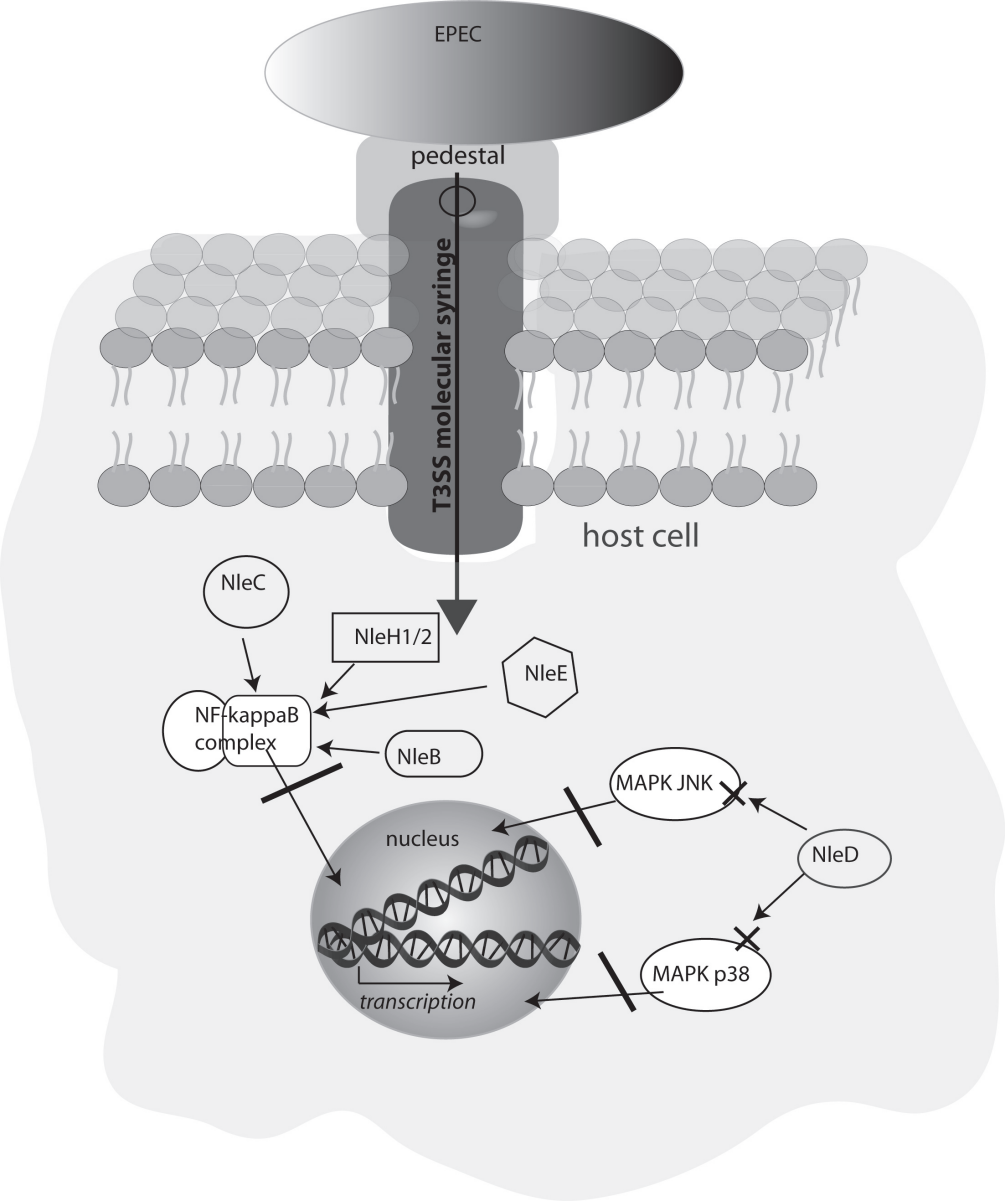
**Proinflammatory signaling pathways are a main target of EPEC effector proteins**. The effectors NleC, NleH1, NleH2, NleE and NleB have been identified to target the NF-kappaB complex at different levels which eventually prevents the nuclear translocation of the p65 subunit. NleD degrades the MAP kinases JNK and p38 which results in impaired signal transduction *via *these pathways. By affecting these crucial inflammatory pathways, EPEC actively impairs the cell to respond to the bacterial stimulus.

The modulation of the host immune response by effectors from *Shigella, Salmonella*, and *Yersinia *is increasingly well understood. Detailed reviews on immune modulation by these pathogens have previously been published elsewhere [30,47]. EPEC, EHEC and *C. rodentium *pathogens have a common set of T3SS effectors composed of seven LEE and a few non-LEE encoded effectors like NleE, NleB, and NleH and show diversity in the repertoire of other non-LEE encoded effectors. The reference strains EHEC 0157:H7 Sakai, EPEC O127:H6 strain E2348/69, EPEC O111:NM strain B171, and *Citrobacter rodentium *have a total of 50, 21, 28 and 29 full length effector genes, respectively [48]. Surprisingly, despite almost 20 years of research on the function of EPEC and EHEC effectors, manipulation of immune defenses in the gut had not been reported until recently; perhaps because as a pathogen which adheres to the surface of epithelial cells, it was not thought to come into contact with host immune cells. The described functions of effectors were mainly the modification of the host cell cytoskeleton in relation to the formation of intestinal attaching/effacing (A/E) lesions and the modification of epithelial tight-junctions in relation to the alteration of intestinal permeability observed during infection [49]. Some apparently contradictory results have been published concerning the pro-or anti-inflammatory activity of EPEC and EHEC. Earlier work demonstrated that the bacteria trigger a pro-inflammatory response [50,51]. However, many studies using epithelial cell lines now clearly show that whereas the bacteria induce an inflammatory response with the detection systems of the host cells, they are able to inhibit the inflammatory pathways in a T3SS-dependent manner [52,53]. By hampering the pro-inflammatory response of epithelial cells, EPEC and EHEC are likely to gain the advantage of reduced cytokine and chemokine secretion which subsequently reduces the recruitment of neutrophils into the affected site. Neutrophils are effective at killing bacteria and release a variety of anti-microbial factors; thus a reduced number and activation of these cells would prolong colonization [54]. Only very recently, anti-inflammatory activity has been demonstrated for the EPEC and EHEC effectors NleE, NleB, NleH, NleD and NleC (Figure 3 and table 1).

NleH1 was the first effector reported to inhibit NF-kappaB in HeLa and HEK293T cell lines [55]. NleH1 and NleH2 both bind the human ribosomal protein S3 (RPS3) in the cytoplasm of infected cells. RPS3 is a non-Rel NF-kappaB subunit and interacts with p65 to increase the transcription of various pro-inflammatory genes [56]. NleH1, but not NleH2, blocks the transcription of RPS3/NF-kappaB-dependent genes by preventing the nuclear translocation of RPS3 [55]. In a gnotobiotic piglet infection model, animals infected with EHEC mutated for *nleH1 *died more rapidly compared to piglets infected with the wild-type or an *nleH2 *mutant. The hypervirulent phenotype that was caused by the nleH1 mutant, seemed to be due to a pronounced inflammatory response [55]. NleHs are auto-phosphorylated serine threonine kinases and share homology with the *Shigella *effector OspG which is known to inhibit NF-kappaB [57]. The RPS3-mediated inhibition of inflammation differs from OspG activity and is independent of the kinase function [55]. It was recently demonstrated that both NleH1 and NleH2 could inhibit NF-kappaB in a kinase-dependent manner [58]. NleH1 and NleH2 prevent TNFalpha-mediated NF-kappaB activation by inhibiting IkappaBalpha ubiquitination and degradation and this activity is dependent on lysine residues present in the kinase domain of both effectors [58]. Streptomycin treated mice infected with EPEC mutated for NleH1 and NleH2 were less colonized, showed higher number of neutrophil infiltration and higher serum level of KC (the mice homologue of IL-8) compared to mice infected with the wild-type, suggesting that NleH promotes colonization and is required for the modulation of the host inflammatory response [58].

NleB is encoded on the same pathogenicity island as NleE in EPEC and EHEC O157 strains, on the integrative element IE6 and the O-island 122, respectively [59]. Presence of the O Island 122 (OI122) is associated with EHEC outbreaks and the hemolytic uremic syndrome, a severe complication of an EHEC infection [60]. *Citrobacter rodentium *strains that have a mutated NleB effector, show an impaired ability to colonise the murine intestine and fail to induce intestinal crypt hyperplasia in the mouse model of infection [45,60]. NleB specifically inhibits NF-kappaB in response to TNFalpha stimulation in epithelial cell lines [61]. NleB's mode of action has yet to be identified but is supposed to act upstream of the IKK complex in the TNFalpha pathway, as NleB is unable to prevent NF-kappaB activation in cells following stimulation with IL-1beta or by bacterial PAMPs [61].

Four independent studies have reported that NleC is a metalloprotease that degrades the p65 NF-kappaB subunit in epithelial cell lines and contributes to the overall anti-inflammatory activity of both EPEC and EHEC strains [43,62-64]. NleC carries the zinc metalloprotease motif 183HEIIH187 which is essential for the proteolytic activity on p65. In addition to p65, NleC also cleaves the NF-kappaB p50 subunit and IkappaBalpha [62,63]. Mülhen and co-workers showed that the N-terminal motif between amino acid 33 and 64 is required for binding to p65 and p50 [62].

EPEC and EHEC produce another zinc metalloprotease, NleD, which specifically degrades MAPK JNK and p38 and contributes to the overall inhibition of IL-8 chemokine secretion by infected cells [43]. Mutation of the zinc metalloprotease motif in NleD, HEXXH, abolishes JNK cleavage.

Colonization and pathogenicity of bacteria mutated for NleC or NleD was not impaired in mice, lamb and calve infection models. Therefore the importance of each of the anti-inflammatory effectors remains to be identified [44-46]. As suggested by *in vitro *data which show that full IL-8 inhibition was dependent on the conjugated activity of mainly NleE and NleC, but also NleB and NleD, it is likely that the mutation of any of the effectors is compensated *in vivo *by the activity of the other [43,63,64]. It would be of interest to test the *in vivo *pathogenicity of a strain deleted for all effectors targeting NF-kappaB to assess the importance of the anti-inflammatory activity for bacterial colonization and persistence.

NleE is a T3SS effector conserved among EPEC, EHEC and *Citrobacter rodentium *strains. NleE is homologous to OspZ which is present in *Shigella *spp strains [65]. Different roles for colonization and persistence have been reported for NleE in *Citrobacter rodentium *mice models of infection [45,66]. Proteomic analysis of cell free *Citrobacter rodentium *secretion profile indicated that NleE, along with EspF and Tir, is the highest secreted effector, suggesting it plays a key role in virulence [67]. Indeed, NleE was shown to be a potent inhibitor of NF-kappaB which prevents nuclear p65 translocation in epithelial cells in response to TNFalpha and IL-1beta [61,68]. The mechanism by NleE blocks NF-kappaB signaling is not known. It was, however, suggested that NleE targets the IKK complex and prevents the phosphorylation of IKKbeta [68]. Although so far no functional domain was found that could explain NleE's mode of action, an analysis of the *nleE *sequence identified a motif 206IDSYMK214 of unknown function which is not sufficient but essential for NleE's anti-inflammatory activity [61].

While other research on NleE showed its inhibiting effect on NF-kappaB signaling in epithelial cells, our group provided evidence that NleE inhibits the expression and production of pro-inflammatory cytokines IL-8, TNFalpha, and IL-6 in human DCs which was due to impaired NF-kappaB p65 nuclear translocation [69]. NleE injected by EPEC was shown to drastically reduce the production of these cytokines in human monocyte-derived DCs as well as PP DCs *in vitro*. We further showed that EPEC injects its effectors into DCs that reach through an epithelial layer in a transwell system. These results suggest that EPEC can impair NF-kappaB signaling not only in epithelial cells, but also hampers the inflammatory response in the gut by injecting into PP DCs that sample the bacteria from the lumen. Certain EPEC strains target the FAE early on in infection [70]. The fact that EPEC can impair signaling in PP DCs when they encounter them at the FAE might explain this phenomenon.

### Preventing phagocytosis

Phagocytosis is a receptor-mediated process and occurs in two different ways: *via *the direct binding of the particle to specific receptors at the surface of the phagocyte or *via *earlier opsonisation of the particle by IgG or the C3bi complement fragment [71]. IgG and C3bi subsequently bind to the FcgammaR or Complement receptor 3 (CR3), respectively, at the surface of the cell. EPEC inhibits phagocytosis in infected macrophages [72]. Furthermore, EPEC blocks both the opsonin-dependent and independent phagocytic pathways *in vitro *by injecting the four T3SS effectors EspF, EspB, EspJ, and EspH.

EspF from both EPEC and EHEC prevents phagocytosis by macrophages and the uptake by M cells in *in vitro *models (table 1) [73-77]. EspF is a multifunctional effector implicated in various others aspect of pathogenesis. Amongst them are the alteration of the intestinal epithelial tight-junctions, the effacement of the brush border microvilli, mitochondrial-dependant apoptosis, the nucleolar disruption and the targeting of various cellular proteins like the neuronal Wiskott-Aldrich syndrome protein (N-WASP), cytokeratin 18, anti-apoptotic Abcf2 or sorting nexin 9 (Snx9), a protein involved in vesicles trafficking [78]. The mechanism by which EspF prevents phagocytosis is still unknown and a study by Quitard and coworkers showing that the N-terminal 101 amino acid domain of EspF is essential suggests that the binding to proteins like N-WASP, actin or SNX9 are not required to prevent the uptake by macrophages [78]. Contradictory to its anti-phagocytic activity, a role for EspF in promoting enterocyte invasion has recently been described and was shown to depend on the interaction between EspF and SNX9 [79]. EspJ from EPEC and EHEC does not block phagocytosis of non-opsonized bacteria but prevents both the FcgammaR and CR3 opsonin-dependant phagocytosis of particles by macrophages or FcgammaR- or CR3-transfected cells [75]. The mechanism by which EspJ blocks opsonophagocytosis remains to be identified. EspB hampers phagocytosis by binding and inhibiting host myosin functions which are required for phagocytosis of non-opsonized bacteria [74]. EspH is the only effector reported to inhibit both opsono- and non-opsonophagocytosis. It binds to the DH-PH domain of several Rho GTPase exchange factors (RhoGEF), preventing activation of Rho GTPases and inducing a general inhibition of actin polymerisation which would explain the inhibition of phagocytosis [73]. The identification of anti-phagocytic activity of so far four effectors translocated by EPEC and EHEC suggest their importance for the *in vivo *pathogenesis. A recent paper reported that the effector EspG targets ARF6 GTPases which results in the reprogramming of endomembrane trafficking [80]. This finding raises the question whether EspG also plays a role in the general anti-phagocytic activity as ARF6 is essential for FcgammaR-mediated phagocytosis [81].

Although EPEC is a non-invasive pathogen, its mucosal uptake has been reported in various *in vitro *and *in vivo *studies [82]. The relevance of EPEC invasion for pathogenesis is not known but might play a role in persistence of the pathogen inside the host. Since EPEC and EHEC mediate disease from their luminal position, one might wonder how relevant the interaction of the bacteria with professional phagocytic cells is during infection. EPEC is known to target the FAE in the gut [70], so the inhibition of its own uptake, which was observed under *in vitro *conditions [76], could reflect the inhibition of its M cells transcytosis resulting in immune evasion. On the other hand, *in vivo *studies with *Citrobacter rodentium *in mice have shown that the activity of neutrophils were necessary to clear the infection, suggesting that at some point during the infection the bacteria interact with phagocytic cells [83].

## Conclusion

Pathogenic bacteria have evolved alongside their hosts, developing sophisticated mechanisms and effector proteins to manipulate the host cells on multiple levels. Not only do bacteria which use the T3SS secure their attachment to epithelial cells by altering the cytoskeleton, they also actively prevent phagocytosis in the gut and impair the immune response by interfering with pro-inflammatory signaling pathways. While these modulatory strategies might not be clinically detrimental to infected individuals, the bacteria gain the advantage of facilitated and prolonged colonization in the gut. The variety of immuno-modulatory effectors in T3SS-employing pathogens might also explain why e.g. EPEC shows a tropism for the GALT, since this is the site where the bacteria encounter DCs which they modulate to hamper the initiation of the intestinal immune response.

## Abbreviations

DC: dendritic cell; EHEC: enterohaemorrhagic *E. coli*; EPEC: enteropathogenic *Escherichia coli*; FAE: follicle-associated epithelium; GALT: gut-associated lymphoid tissue; PP: Peyer's patch; T3SS: type-3 secretion system.

## Competing interests

The authors declare that they have no competing interests.

## Authors' contributions

All authors wrote, read and approved the final manuscript.
